# Gut Microbiota Influence Lipid Metabolism of Skeletal Muscle in Pigs

**DOI:** 10.3389/fnut.2021.675445

**Published:** 2021-04-13

**Authors:** Choufei Wu, Wentao Lyu, Qihua Hong, Xiaojun Zhang, Hua Yang, Yingping Xiao

**Affiliations:** ^1^Key Laboratory of Vector Biology and Pathogen Control of Zhejiang Province, School of Life Sciences, Huzhou University, Huzhou, China; ^2^State Key Laboratory for Managing Biotic and Chemical Threats to the Quality and Safety of Agro-Products, Institute of Agro-Product Safety and Nutrition, Zhejiang Academy of Agricultural Sciences, Hangzhou, China; ^3^College of Animal Sciences, Zhejiang University, Hangzhou, China; ^4^Institute of Animal Husbandry and Veterinary Medicine, Jinhua Academy of Agricultural Sciences, Jinhua, China

**Keywords:** gut microbiota, fecal microbiota transplantation, lipid metabolism, intramuscular fat, pig

## Abstract

Gut microbiota is recognized as a strong determinant of host physiology including fat metabolism and can transfer obesity-associated phenotypes from donors to recipients. However, the relationship between gut microbiota and intramuscular fat (IMF) is still largely unknown. Obese Jinhua pigs (JP) have better meat quality that is associated with higher IMF content than lean Landrace pigs (LP). The present study was conducted to test the contribution of gut microbiota to IMF properties by transplanting fecal microbiota of adult JP and LP to antibiotics-treated mice. Similar to JP donors, the mice receiving JP's microbiota (JM) had elevated lipid and triglyceride levels and the lipoprotein lipase activity, as well as reduced mRNA level of angiopoietin-like 4 (ANGPTL4) in the gastrocnemius muscles, compared to those in mice receiving LP's microbiota (LM). High-throughput 16S rRNA sequencing confirmed that transplantation of JP and LP feces differently reconstructed the gut microbiota in both jejunum and colon of mouse recipients. In colonic samples, we observed an elevated ratio of Firmicutes to Bacteroidetes and increased abundance of genus *Romboutsia* in JM, which were positively correlated with obesity. Furthermore, the abundance of *Akkermansia* decreased in JM, which is positively correlated with lean. Colonic concentrations of acetate (*P* = 0.047) and butyrate (*P* = 0.014) were significantly lower in JM than in LM, and consistently, the terminal genes for butyrate synthesis, butyryl CoA: acetate CoA transferase were less abundant in colonic microbiota of JM. Taken together, these gut microbiota of obese JP intrinsically promotes IMF accumulation and can transfer the properties to mouse recipients. Manipulation of intestinal microbiota will, therefore, have the potential to improve the meat quality and flavor of pigs and even to ameliorate the metabolic syndrome in human.

## Introduction

China has the largest pork industry in the world. Growth rate, meat quality, and meat flavor are economically important in pig production that influence consumer acceptance (1, 2). In the past few decades, increasing lean meat content and reducing backfat thickness were the main targets of pig breeding (3). However, improvement of the sensory properties and nutritional value of pork has become a priority in recent years (4, 5), which is closely related to fatness traits such as intramuscular fat (IMF) content (6). Many studies have suggested that IMF have positive correlations with pork tenderness, juiciness, shearing force, and taste (7–9). Chinese local breeds such as Jinhua pig (JP) have distinctively higher IMF content and better meat quality than the introduced pig breeds, such as the Landrace (LP), which is a lean-type breed characterized by a fast growth rate and high lean meat content (10, 11). As muscles with sufficient IMF content are particularly suitable for conversion to dry products, JP is the excellent raw material of Jinhua-Ham, one of the most famous brands in China (10).

The genetic basis of IMF contents across multiple pig breeds has been investigated in several researches (10, 12, 13). However, the fact that the average heritability of IMF in the literature is only about 0.5 (ranging from 0.21 to 0.86) (14) suggests that alternative mechanisms for this trait in pigs may exist. As a forgotten organ in mammals that contains more genes than mammalian genome and that add a broad range of biological functions that the host could not otherwise perform, the gut microbiota has been proved to be a major contributor of obesity-associated phenotypes, as the propensity for adipogenesis can be transferred from donors to recipients through fecal microbiota transplantation (FMT) (15–19). There are also a few lines of evidence showing that the depletion of gut microbiota leads to increased muscle fatty acid catabolism (20) and ingestion of probiotics/prebiotic influences the skeletal muscle development and metabolic profile (21, 22). Moreover, the skeletal muscle properties are transmissible via FMT (23). These findings suggest a link between the gut microbiota composition and fat deposition in skeletal muscle.

One postulated mechanism underlying the interactions between gut microbiota and host fat metabolism relates to the regulation of lipoprotein lipase (LPL), a key enzyme in lipid metabolism by modulating intestinal epithelial expression of angiopoietin-like protein 4 (ANGPTL4/fasting-induced adipose factor, FIAF), a circulating LPL inhibitor susceptible to gut microbiota (24–26). Another mechanism involves the production of short-chain fatty acids (SCFAs), the major fermentation products of undigestible carbohydrates that are available to the gut microbiota. They are rapidly absorbed and utilized by the host and elicit effects on lipid metabolism and adipose tissue at several levels (27). LPL and SCFAs have also been associated with the IMF contents (28–31), while their functions in gut microbiota-mediated intramuscular fat metabolism remain unclear.

We have recently revealed significantly different gut microbiota structures between Jinhua pigs (a slow-growing breed with a high propensity for adipogenesis) and Landrace pigs (a lean, fast-growing breed with the high carcass yield), and found that gut microbiota plays an important role in contributing to adiposity in pigs (11, 19, 32). In the present study, we further compared the propensity for IMF accumulation between the two pig breeds and identified the contribution of gut microbiota by transplanting their respective fecal microbiota to antibiotic-treated mice, and the gut microbial community structure and IMF contents were monitored in mouse recipients. Our study uncovered a critical role of the gut microbiota in regulating the fat metabolism of skeletal muscle and provided a better understanding of the molecular mechanism.

## Methods

### Animals

Pigs: Ten Jinhua and 10 Landrace pigs, with 5 males and 5 females of similar weights in each breed, were housed in the same environmentally controlled room in a swine breeding farm and fed a regular commercial corn-soybean diet. At 240 days of age, spontaneously excreted feces were collected freshly from each animal, mixed in equal amounts within the same breed to generate a “representative” fecal material for each breed.

Mice: A total of 24 28-day-old C57BL/6J mice (12 male and female each) were maintained in gnotobiotic isolators in SPF Animal Technology Co. (Beijing, China) under a strict 12-h light/dark cycle, and fed an autoclaved chow diet *ad libitum*.

### Fecal Microbiota Transplantation

The stool suspension was then prepared as previously described (19). In brief, the fecal samples were diluted 5-fold (v/w) and homogenized in sterile pre-reduced phosphate buffer (PBS, 0.1 mol/L, pH7.2). After being thoroughly mixed and standing for 1 min, the supernatant was withdrawn and stored at −80°C in aliquots until used as the fecal inoculum.

Prior to fecal microbiota transplantation, the intestinal commensal bacteria was first depleted by the administration of an antibiotic cocktail (0.5 g/L vancomycin, 1 g/L neomycin sulfate, 1 g/L metronidazole, 1 g/L ampicillin) in drinking water ad lib according to our previous procedures (19). After 28 days of continuous treatment, the mice were randomly divided into 2 groups (12 in each group, half male and half female) and infused by intragastric gavage with fecal suspension of Jinhua or Landrace pigs respectively. The dosage was 0.2 mL per mouse once daily for 7 days. The mice were maintained for another 28-day after inoculation.

### Sample Collection of Donors and Recipients

The gastrocnemius muscles (GM) from the pigs (*n* = 10 per breed) and mice (*n* = 12 per group) were sampled at euthanasia for RNA extraction and biochemical measurements. The jejunum and colon contents were obtained for 16S rRNA gene sequencing, qPCR, and SCFA analysis.

### Quantitative PCR (qPCR)

Total RNA from the GM was isolated with RNeasy Plus Mini kit (Qiagen) and reverse-transcribed to synthesize cDNA using SuperScript II Reverse Transcriptase (Invitrogen) according to the manufactures' instructions. The cDNA library of each sample was then subjected to qPCR reactions in triplicate on an ABI Prism 7700 Detection system (Applied Biosystems, Foster City, CA, USA) using an annealing temperature of 63°C. The primers for LPL, ANGPTL4 and the internal standard of GAPDH were listed in Table 1. Data were normalized to GAPDH or 18S rRNA and calculated by the 2-ΔΔCT method (33).

**Table 1:** Primers of the target genes for pig and mouse used in RT-qPCR.

**Species**	**Gene**	**Genbank Accession**	**Primer sequences(5^**′**^ to 3^**′**^)**	**Size (bp)**
Pig	LPL	NM_214286.1	CCCTATACAAGAGGGAACCGGAT	138
			CCGCCATCCAGTCGATAAACGT	
	ANGPTL4	NM_001038644.1	CGACCTCCGAGGAGACAAGAA	108
			CGAGGGATGGAATGGAAGTACTG	
	GAPDH	AF017079	GGCAAATTCCACGGCACAGTCA	82
			CTCGCTCCTGGAAGATGGTGAT	
	18S	NR_046261	GCCCTATCAACTTTCGATGGTAGTC	113
			CCTTGGATGTGGTAGCCGTTTCTCA	
Mouse	LPL	NM_008509.2	CCAAGCTGGTGGGAAATGATGTG	95
			GCTGTACCCTAAGAGGTGGACGTT	
	ANGPTL4	NM_020581.2	CCTACAAGGATGGCTTCGGAGAT	86
			GCTTCCTCGGTTCCCTGTGAT	
	GAPDH	GU214026.1	CAGTATGACTCCACTCACGGCAA	100
			CTCGCTCCTGGAAGATGGTGAT	
	18S	NR_003278	CGGACACGGACAGGATTGACA	94
			CCAGACAAATCGCTCCACCAACTA	

### Measurement of IMF Content

The IMF contents were measured using the Soxhlet method according to our previous study (32).

### Biochemical and Enzymatic Measurements

GM samples (~100 mg) were homogenized in 1 ml of ice-cold PBS. After centrifugation at 2500 rpm for 10 min at 4°C, the supernatant was decanted for measuring LPL activity and the triglyceride content with a LPL assay kit and a triglyceride assay kit (Jiancheng Bioengineering Ltd, Nanjing, China) according to the manufacturer's instructions, respectively. The LPL activity was expressed as U/mg protein of muscle tissue, and the triglyceride content was expressed as mmol/g protein of muscle tissue.

### DNA Extraction and Microbiota Analysis

The genomic DNA was extracted from the jejunal and colonic contents of mice using QIAamp DNA Stool Mini Kit (QIAGEN, Valencia, CA, USA). The 16S rRNA gene sequencing and data analysis were performed as described previously (34). Briefly, the V3 and V4 region of bacterial 16S rRNA gene was amplified from each genomic DNA sample by using the barcode-fusion primers 341F and 806R. The DNA libraries were then constructed using TruSeq DNA PCR-Free Library Preparation Kit (Illumina) and sequenced on an Illumina MiSeq platform. To obtain clean sequencing data, the chimeric reads were identified and removed by using USEARCH. Operational taxonomic units (OTUs) were assembled at 97% sequence similarity. A representative sequence was picked for each OTU and annotated with taxonomic information using the RDP classifier (35). Pie charts showing taxa distribution at the phylum and genus levels were constructed. Principal coordinate analysis (PCoA) was conducted to illustrate the β-diversity based on weighted UniFrac distances.

### Analysis of Short-Chain Fatty Acids

The concentrations of SCFAs were measured by gas chromatography (GC) using the method as described in our previous report (36). Briefly, 0.1 g of the colonic contents were vortex-mixed vigorously with 10 mL deionized water. After the mixture were centrifuged (12,000 rpm for 10 min), 500 μL aliquots of the supernatant were added to 100 μL of 25% (w/v) metaphosphoric acid and crotonic acid (internal standard). The mixed solution was filtered with a 0.22 μm mesh and was then employed to measure the concentrations of SCFAs by GC (GC-2010 plus, Shimadzu, Kyoto, Japan).

### qPCR Analysis of Key Bacteria and Genes in Butyrate Production

The abundances of the major butyrate-producing bacteria, clostridial cluster I, IV, and XIVa and the terminal genes for butyrate synthesis, butyrate kinase (BK) and butyryl CoA: acetate CoA transferase (BCoAT) in mice colon contents were assessed by qPCR on an ABI Prism 7700 detection system (Applied Biosystems, Foster City, CA, USA) using the extracted DNA as templates and SYBR Green PCR Master Mix (Takara, Japan), as previously described (34, 37). DNA was amplified under the following conditions: 95°C for 2 min, and 35 cycles of 15 s at 95°C, 45 s at 58°C, and 1 min at 72°C. Each sample was analyzed in triplicate. The primer sets used were listed in Table 2. All qPCR results were expressed as gene copies per g of colon contents.

**Table 2:** Primers of key bacteria and genes in butyrate production used in qPCR analysis.

**Item**	**Primers (5^′^ → 3^′^)**
Clostridial cluster I	F:TACCHRAGGAGGAAGCCAC
	R:GTTCTTCCTAATCTCTACGCAT
Clostridial cluster IV	F:ATGCAAGTCGAGCGA(G/T)G
	R:TATGCGGTATTAATCT(C/T)CCTTT
Clostridial cluster XIVa	F:CGGTACCTGACTAAGAAG
	R:AGTTT(C/T)ATTCTTGCGAAC
Butyryl-CoA acetate-CoA transferase	F: AAGGATCTCGGIRTICAYWSIGARATG
	R:GAGGTCGTCICKRAAITYIGGRTGNGC
Butyrate kinase	F:TGCTGTWGTTGGWAGAGGYGGA
	R:GCAACIGCYTTTTGATTTAATGCATGG

### Statistical Analysis

Data are expressed as means ± SEM. All statistical analyses were performed using SPSS (SPSS, Chicago, IL, United States). Unpaired two-tailed Student's *t*-test was used to evaluate the differences between two groups. A *P*-value < 0.05 was considered a significant difference.

## Results

### Jinhua Pigs Were More Efficient in IMF Deposition Than Landrace Pigs

**Figure 1:**
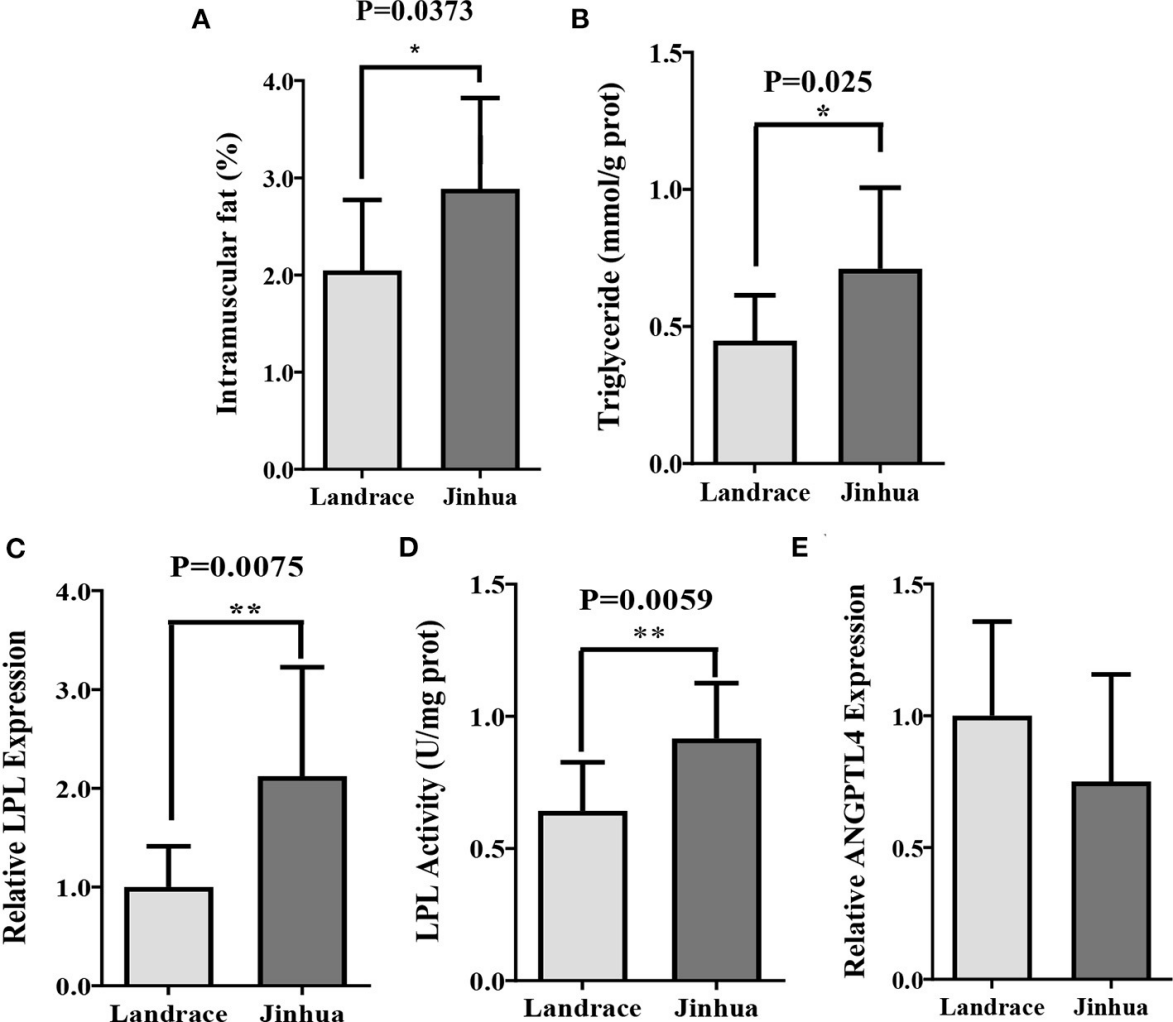
Intramuscular fat deposition in Landrace and Jinhua pigs. Intramuscular fat **(A)**, triglyceride **(B)**, relative lipoprotein lipase (LPL) expression **(C)** and activity **(D)**, and relative ANGPTL4 expression **(E)** in gastrocnemius muscle of Landrace and Jinhua pigs. The results were shown as means ± SEM of 10 pigs. *P < 0.05; **P < 0.01.

To observe the differences in intramuscular fat (IMF) metabolism between the Jinhua and Landrace pigs, the two breeds were sacrificed at 240 days of age and the gastrocnemius muscles (GM) were sampled. As shown in Figures 1A,B, the Jinhua pigs had significantly higher level of intramuscular fat (*P* = 0.0373) and triglycerides (*P* = 0.025) than the Landrace pigs. Consistently, both the expression level (*P* = 0.0075) and activity (*P* = 0.0059) of lipoprotein lipase (LPL, Figures 1C,D) were elevated in GM samples of Jinhua pigs (Figure 1E). These findings were in accordance with the fact that Jinhua pigs are more efficient in IMF deposition than Landrace pigs.

### Mouse Recipients Resembled Their Respective Pig Donors in IMF Deposition

**Figure 2:**
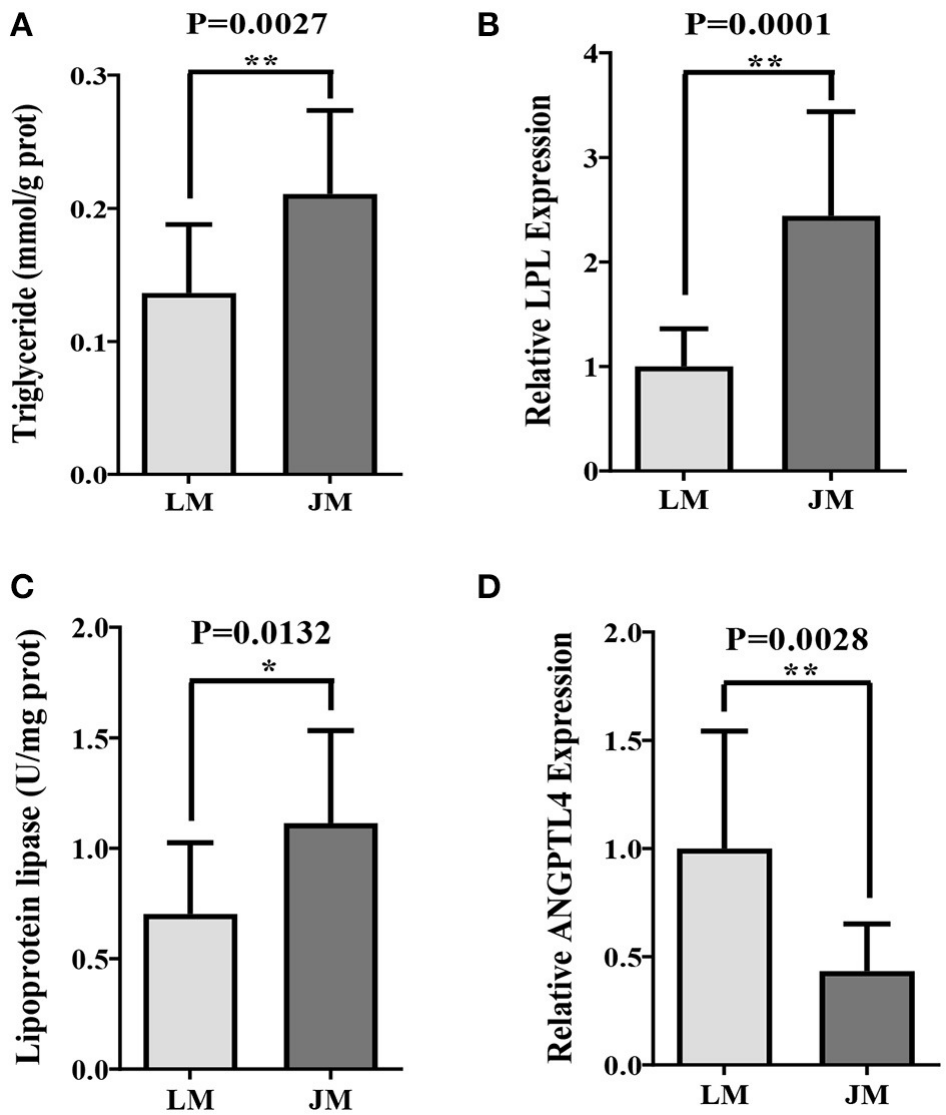
Intramuscular fat deposition in mice receiving fecal microbiota of Landrace and Jinhua pigs. Intramuscular triglyceride **(A)**, relative lipoprotein lipase (LPL) expression **(B)** and activity **(C)**, and relative ANGPTL4 expression **(D)** in gastrocnemius muscle of mouse recipients. JM, mice receiving fecal microbiota from Jinhua pigs; LM, mice receiving fecal microbiota from Landrace pigs. *P < 0.05; **P < 0.01.

To elucidate whether the IMF metabolic profiles were also affected by gut microbiota, FMT was carried out and triglyceride content, LPL expression and activity, as well as ANGPTL4 mRNA level in GM of mouse recipients were examined. Remarkably higher intramuscular levels of triglyceride content and LPL expression and activity in mouse recipients of Jinhua's feces (JM) than those in mouse recipients of Landrce pigs' feces (LM) were observed (Figures 2A–C). Furthermore, the ANGPTL4 mRNA expression level in JM was shown to be correspondingly was decreased compared to LM (Figure 2D). Collectively, the mouse recipients exhibited similar characteristics in IMF metabolism as their respective pig donors, suggesting that gut microbiota is capable of influencing and transferring the IMF trait across species.

### Gut Microbiota of Mouse Recipients Were Differently Reconstructed by FMT From Jinhua and Landrace Pigs

**Figure 3:**
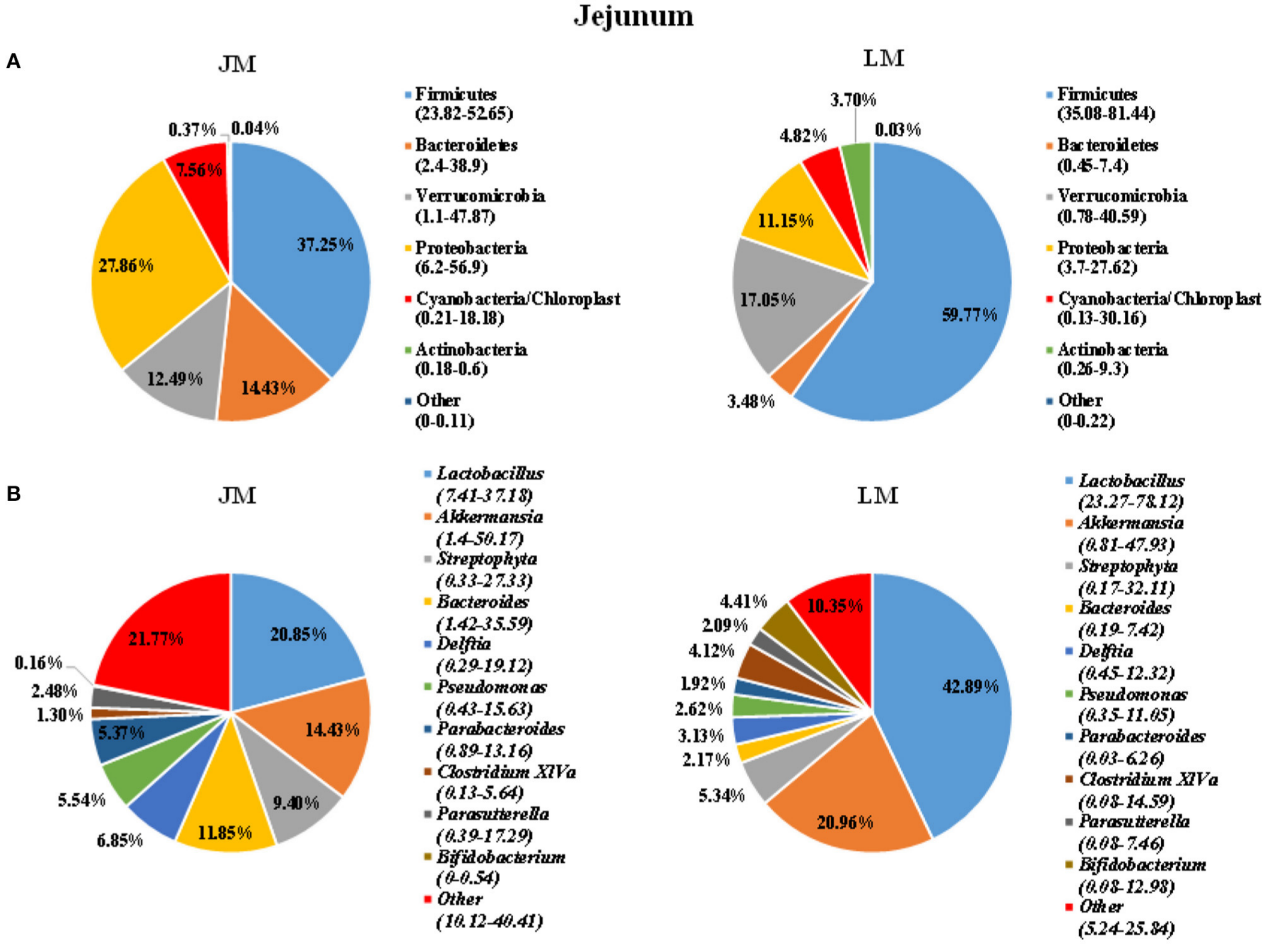
Pie charts showing taxa distribution of bacterial community in jejunum of mouse recipients at the phylum **(A)** and genus **(B)** levels. Top 6 phyla and top 10 genera are shown.

**Figure 4:**
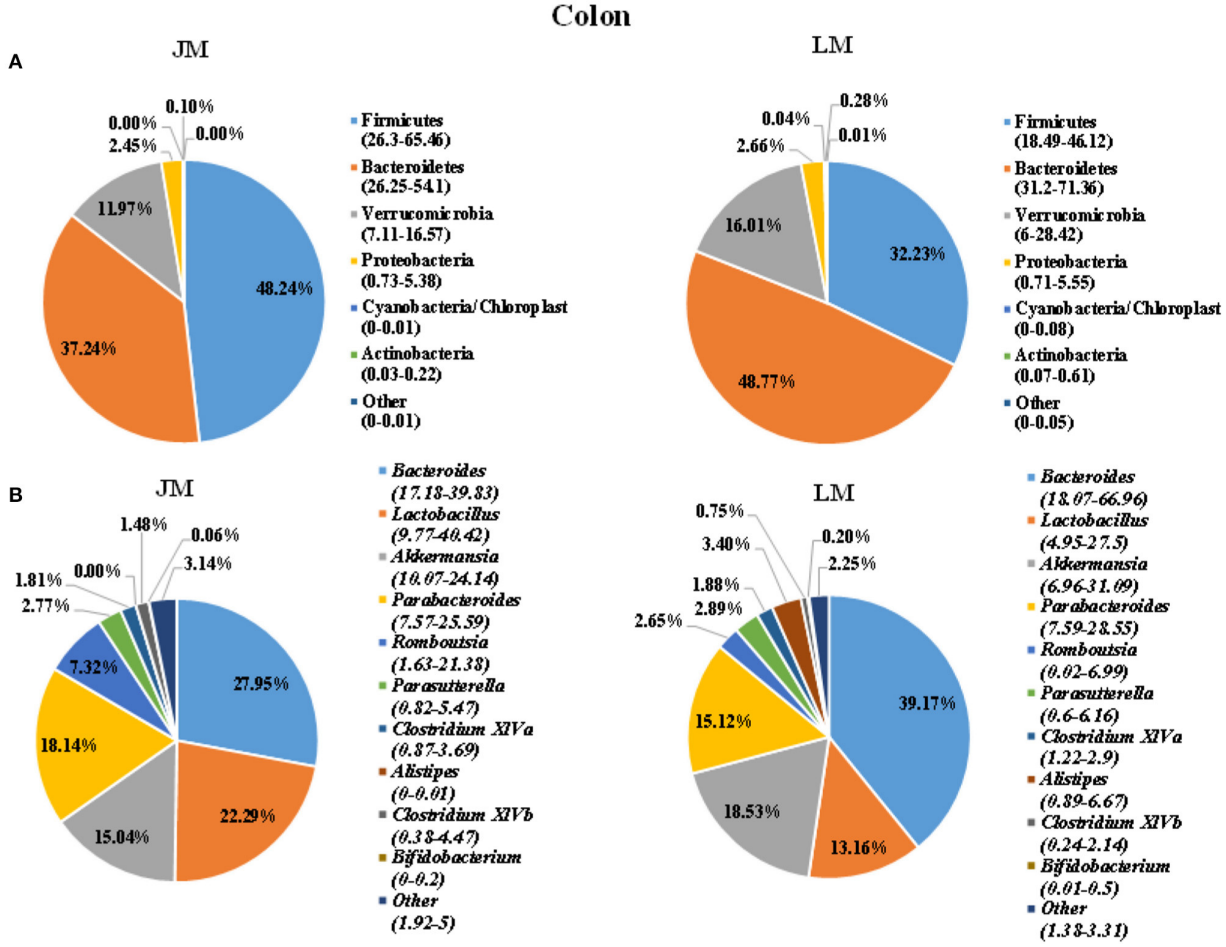
Pie charts showing taxa distribution of bacterial community in colon of mouse recipients at the phylum **(A)** and genus **(B)** levels. Top 6 phyla and top 10 genera are shown.

To identify the gut microbiota throughout the small and large intestine in the two groups of mouse recipients, the jejunal and colonic contents were obtained from individual mice and subjected to 16S rRNA gene sequencing. A total of 2,243,174 clean reads with an average length of 418 bps were generated from all samples, which were further grouped into 682 OTUs at the 97% identity level, with an average OTU number of 173 per sample (range = 103 ~ 355, SEM = 60.70). Taxonomic analysis showed that Firmicutes, Bacterioidetes, Verrucomicrobia, and Proteobacteria were the most abundant phyla in both jejunum and colon, accounting for more than 90% of the total sequences in most samples (Figures 3, 4). The ratio of Firmicutes to Bacteroidetes that was associated with the obesity phenotype was remarkably lower in jejunal samples of JM group than in those of LM group (Figure 3A), while it was elevated in colonic samples of JM group as compared to those of LM group (Figure 4A). Verrucomicrobia was greatly less abundant in both jejunum and colon of JM group as compared to LM group, while Proteobacteria was more prevalent in the jejunum of JM group than in LM group.

At the genus level, Lactobacillus, Akkermansia, Streptophyta, Bacteroides, and Clostridium XIVa were among the most abundant genera in the jejunum (Figure 3). Lactobacillus and Akkermansia made up larger proportions in the jejunum of LM group than in JM group, while Streptophyta and Bacteroides were more enriched in JM group. Notably, Clostridium XIVa, a main genus of butyrate-producing bacteria was remarkably reduced in the jejunum of JM (Figures 3A,B). In colon samples, Bacteroides, Lactobacillus, Akkermansia, Parabacterioides, and Rombontsia constituted the top five genera in both groups of mice. Among them, Bacteroides and Akkermansia decreased while Lactobacillus, and Rombontsia increased in the colon of JM group compared to LM (Figure 4B).

**Figure 5:**
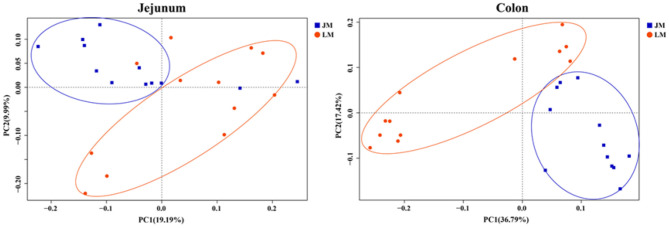
Principal coordinates analysis (PCoA) of the jejunum and colon bacterial community composition of mouse recipients based on unweighted unifrac distance.

To evaluate the degree of discrepancy between the bacterial community structures of mouse recipients, a principal coordinate analysis (PCoA) was performed. As delineated in Figure 5, the microbiota in the jejunum and colon were significantly different by both PCoA1 and PCoA2. They were further separated according to their respective donors by PCoA1 (for jejunal samples) or both coordinates (for colonic samples), suggesting that transplantation of Landrace and Jinhua feces differently reconstructed the gut microbiota in both small and large intestines of mouse recipients.

### Colonic Concentrations of Acetate and Butyrate Were Lower in JM

To investigate the difference of SCFA in the mouse recipients, the colonic concentrations of acetate, propionate, butyrate, iso-butyrate, valerate, and iso-valerate were assessed. The concentrations of acetate (*P* = 0.047) and butyrate (*P* = 0.014), as well as total SCFA (*P* = 0.042) were significantly lower in the colon of JM than in LM, while no differences were observed in propionate, butyrate, iso-butyrate, valerate, and iso-valerate between the two groups (Table 3).

**Table 3:** The concentration of short-chain fatty acids in the colon of mice receiving fecal microbiota of Jinhua and Landrace pigs.

**Items**	**JM**	**LM**	**SEM**	***P*-value**
Acetate	1.84	2.37	0.21	0.047
Propionate	0.44	0.41	0.11	0.721
Butyrate	0.30	0.52	0.08	0.014
Iso-butyrate	0.22	0.25	0.07	0.589
Valerate	0.18	0.10	0.09	0.314
Iso-valerate	0.16	0.19	0.1	0.263
Total SCFAs	3.14	3.84	0.32	0.042

*The concentrations of SCFAs were expressed as mg/g of fresh colonic contents*.

### Key Genes in Butyrate Biosynthesis Were Less Abundant in Colonic Microbiome of JM

Based on the observation that butyrate was remarkably decreased in the colon of LM, we further examined the abundances of the major butyrate-producing bacteria, clostridial cluster I, IV, and XIVa and the terminal genes for butyrate synthesis, butyrate kinase (BK) and butyryl CoA: acetate CoA transferase (BCoAT) in colonic contents of the two groups of mouse recipients by qPCR. As shown in Table 4, although the abundance of clostridial clusters of butyrate-producing bacteria was comparable in between JM and LM, the copies of BK and BCoAT genes were significantly fewer in the colonic samples of JM, consistent with the diminished production of butyrate in JM.

**Table 4:** The abundances of butyrate-producing bacteria and terminal genes for butyrate synthesis in the colon of mice receiving fecal microbiota of Jinhua and Landrace pigs.

**Item**	**JM**	**LM**	**SEM**	***P*-value**
Clostridial cluster I	6.56	7.21	0.93	0.109
Clostridial cluster IV	5.85	6.08	0.52	0.210
Clostridial cluster XIVa	8.15	8.43	1.09	0.662
Butyryl-CoA acetate-CoA transferase	6.21	7.19	0.58	0.041
Butyrate kinase	5.97	6.63	0.71	0.076

*The abundance of bacterial groups was expressed as log10 16S rRNA gene copies/g of fresh colonic contents and of genes related to butyrate synthesis was expressed as log10 gene copies of total DNA/g of fresh colonic contents*.

## Discussion

Over the last decade, extensive research has revealed a critical role of gut microbiota in the physiology of both fat deposition and obesity by affecting host energy harvest and fat metabolism (16–19, 38). There is substantial evidence indicating that skeletal muscle properties including lipid metabolic profile and fiber characteristics are closely linked to the presence of obesity (23, 39). Some researchers suggest that a gut microbiota-muscle axis might exist in the body (40). However, less attention has been paid to the association between the gut microbiota and IMF accumulation.

Jinhua and Landrace pigs display notable differences in the body fat content and propensity for adipogenesis, which make them good models to study human overweight and obesity (32, 41, 42). Our previous study has proved that gut microbiota of obese Jinhua pigs is capable of enhancing adipogenesis and fat deposition than that of lean Landrace pigs, and that the obesity-associated phenotypes is transferrable across species (23). Here, we further demonstrated that Jinhua pig-derived microbiota also enhances IMF content in their mouse recipients (JM) of FMT. Both Jinhua pig and JM exhibited higher triglyceride concentrations and LPL expression and activity in skeletal muscle as compared to LP and LM, respectively. LPL provides fatty acid for tissue utilization and storage and can be inhibited by ANGPTL4 that is susceptible to regulation by gut microbiota and its metabolites, SCFA (24–26). In line with the increased expression and activity of LPL, the expression level of ANGPTL4 in GM was found to be correspondingly decreased in both Jinhua pigs and JM. These findings support an implication of gut microbiota in intramuscular adipogenesis.

The feces and large intestinal contents are often analyzed to indicate the alterations in gut microbiota of FMT recipients. However, the impact of FMT on the microbiota in small intestine is barely known. To clarify that, jejunum microbiota of mouse recipients was analyzed in our present study. Significant differences were found between the two groups of mice. The phyla of Bacterioidetes and Proteobacteria, and the genera of Streptophyta and Bacteroides were more enriched in jejunum samples of JM group, while the phylum of Firmicutes and Verrucomicrobia and the genera of Lactobacillus, Akkermansia and Clostridium XIVa were more abundant in jejunum samples of LM. This data suggested that microbiota in small intestines of mouse recipients were also reconstructed following FMT.

In colonic samples, we observed an elevated ratio of Firmicutes to Bacteroidetes in JM, which may led to the development of obesity in human and rodents (24). Genus Romboutsia that is positively correlated with obesity (43) was also increased in the colon of JM. On the other hand, a significant diminishment of genus Bacteroides, an important SCFA producer, was observed, which was consistent with the reduced level of colonic SCFAs in JM. SCFAs act as substrates or signal molecules, which are transported into blood from the intestinal lumen and subsequently taken up by body organs in the host (44, 45). The SCFA can induce the transcription and secretion of ANGPTL4 in intestinal cells and adipocyte, and the elevations of ANGPTL4 have been reported to be associated with the inhibition of fat deposition (46). Taken together, the modulation of colonic microbiota and the decreased SCFAs generation esp. acetate and butyrate in JM might positively contribute to the intramuscular adipogenesis in mouse recipients.

Some researchers have reported that dietary supplementation of butyrate can prevent diet-induced insulin resistance and obesity by promoting energy expenditure and induce mitochondria function (46, 47) and can decrease IMF content in mice and Broilers (30, 48). Here we found a remarkable decrease of colonically derived butyrate in JM compared with that in LM, which might contribute to the higher IMF content in JM. However, the abundances of the major butyrate-producing bacteria, clostridial cluster I, IV, and XIVa were comparable in the colon between JM and LM as determined by qPCR, perhaps because these clostridial clusters still harbor a diverse collection of non-butyrate producers. Therefore, assessing terminal genes of butyrate synthesis pathways, namely, the butyrate kinase (BK) pathway and the butyryl CoA: acetate CoA transferase (BCoAT) pathway, could be valuable to indicate the activity of the butyrate producer (37, 49). Fewer copies of BK and BCoAT genes were detected in the colon of JM samples by qPCR, suggesting a decreased abundance or activity of butyrate-producing bacterial community and explaining the diminished production of butyrate in JM.

## Conclusion

Our results demonstrated that Jinhua pig has a higher IMF content than Landrace pig and the phenotype could be recapitulated by gut microbiota in respective mouse recipients. The mechanism might be related to the regulation of ANGPTL4 and consequently LPL expression and activity, as well as to the modulation of colonically derived SCFAs. This study has opened possibilities for manipulating the meat quality and the sensory properties of lean commercial pig breeds through modulating the gut microbiota. Moreover, it might provide a model to investigate the link of gut microbiota with the distribution of adipose tissue and deposition of ectopic fat in human.

## Data Availability Statement

The datasets presented in this study can be found in online repositories. The names of the repository/repositories and accession number(s) can be found here: NCBI Sequence Read Archive (SRA) with accession number PRJNA707602.

## Ethics Statement

The animal study was reviewed and approved by the Animal Care and Use Committee of Zhejiang Academy of Agricultural Sciences.

## Author Contributions

CW, YX, and HY designed the experiment. CW, WL, QH, XZ, and YX conducted the animal experiments. CW, HY, and YX wrote and revised the manuscript. CW, QH, HY, and YX did experimental analysis, collected, and analyzed the data. All authors reviewed the manuscript.

## Conflict of Interest

The authors declare that the research was conducted in the absence of any commercial or financial relationships that could be construed as a potential conflict of interest.
